# Single-Cell RNA Sequencing of the Nucleus Pulposus Reveals Chondrocyte Differentiation and Regulation in Intervertebral Disc Degeneration

**DOI:** 10.3389/fcell.2022.824771

**Published:** 2022-02-21

**Authors:** Shuo Han, Yiran Zhang, Xianjuan Zhang, Hao Zhang, Shengwei Meng, Meng Kong, Xiaojie Liu, Xuexiao Ma

**Affiliations:** ^1^ Department of Spinal Surgery, The Affiliated Hospital of Qingdao University, Qingdao, China; ^2^ Department of Medicine, Qingdao University, Qingdao, China; ^3^ Medical Research Center, Shandong Institute of Orthopaedics and Traumatology, The Affiliated Hospital of Qingdao University, Qingdao, China; ^4^ Department of Pathogenic Biology, Qingdao University Medical College, Qingdao, China; ^5^ Department of Spinal Surgery, Qingdao Municipal Hospital, Qingdao, China; ^6^ 970 Hospital of the PLA Joint Logistic Support Force, Weihai, China

**Keywords:** single-cell RNA sequencing, intervertebral disc degeneration, nucleus pulposus, chondrocyte, differentiation and regulation

## Abstract

The nucleus pulposus (NP), a heterogeneous tissue, is an essential functional component of the intervertebral disc. However, NP cell development route and regulation mechanism in intervertebral disc degeneration (IVDD) remain unknown. Here, we performed single-cell RNA sequencing of six NP samples with normal control, mild degeneration, and severe degeneration. Based on unbiased clustering of gene expression patterns from 30,300 single-cell RNA sequencing, we identified three cell lineage families of macrophages, endothelial, and chondrocyte cells and characterized seven chondrocyte subtypes, and defined two developmental pathways of the chondrocyte cell lineage families in the process of IVDD. Additionally, CellPhoneDB analysis revealed potential interactions between chondrocyte cells and other cells in IVDD. Chondrocytes in one of the differentiated orientations interact with macrophages and endothelial cells and have an inflammatory amplification effect, which were key factors causing IVDD. Collectively, these results revealed the dynamic cell landscape of IVDD development and offered new insights into the influence of NP cells differentiation on extracellular matrix homeostasis during degeneration, providing potential treatment targets for IVDD.

## Introduction

IVDD (intervertebral disc degeneration), a persistent age-related disease, has been recognized as a primary source of low back pain (Risbud and Shapiro 2014). The senior population had an IVDD prevalence of more than 90%, resulting in a massive burden on the worldwide healthcare system (Teraguchi et al., 2014; Safiri et al., 2021). The nucleus pulposus (NP), annulus fibrous, and cartilaginous endplate (CEP) of the IVD link adjoining vertebral bodies sustain body load, absorb vertical vibration, and preserve spinal mobility (Humzah and Soames 1988). The NP is a gelatinous substance made up of nucleus pulposus cells (NPCs) and extracellular matrix (ECM). ECM in healthy NP is mostly made up of type II collagen and proteoglycan (Le Maitre et al., 2007). Because of its extremely hydrating characteristics, NP may deform reversibly, which helps to alleviate mechanical conduction and balanced load distribution (Iatridis et al., 1996).

IVDD is defined by changes in the biological structure and function of NP, which are primarily influenced by the phenotype and function of NPCs (Lawson and Harfe 2015; Li et al., 2019). During the development of IVDD, NPCs increased the expression of ECM degrading enzymes, such as matrix metalloproteinases (MMPs) and disintegrin-like and metalloprotease with thrombospondin motifs (ADAMTSs). While the inhibitor protein, tissue inhibitor of metalloproteinases (TIMPs), is not expressed adequately, it fails to buffer the increased catabolism process and preserve ECM homeostasis in NP (Le Maitre et al., 2007; Vo et al., 2013). Cytokines, including interleukin (ILs), tumor necrosis factors (TNFs), and growth factors (GFs), play an important role in regulating NPC activity and attracting inflammatory and endothelial cells (ECs) throughout this process (Risbud and Shapiro 2014; Kwon et al., 2017). The secretion of proteoglycan and type II collagen by NPCs declines as a result of different processes, while the synthesis of type I and III collagen rises, resulting in ECM remodeling, which damages the tissue structure of NP and the ecological balance of IVD (Le Maitre et al., 2007; Yan et al., 2017). As a result, the pathogenic mechanism of IVDD is a complicated network involving many cell connections and dynamic regulatory mechanisms.

The dynamic alterations in the expression profile of NPCs in IVDD development are now well characterized. For example, Risbud et al. (2007) first isolated NPCs with stem/progenitor cell features from IVDD tissues, while Gilson et al. (2010) discovered a notochordal-like cell subpopulation in the NP of adult bovine IVDs. Understanding the variability of NPCs might aid in the design of fundamental strategies for biological and targeted IVDD treatment. Most existing studies, however, have been limited to the bulk level, which fails to explore the heterogeneity of NPCs and their unique roles in IVDD from a high-resolution perspective; additionally, the transcriptional regulation and cellular interactions that contribute to disease progression are unknown.

Single-cell transcriptome sequencing (scRNA-seq) has become increasingly popular in the study of tissue and cell heterogeneity, and its molecular regulatory mechanisms in physiological development, pathological processes, inflammation, and immunity as high-throughput sequencing technology have improved and innovated (Potter 2018). In this study, we performed scRNA-seq of the NP with various degrees of IVDD and explore novel cellular interactions and crucial molecular pathways contributing to the disease development.

## Methods

### Ethical Approval and Consent

The procedures used in this study were approved by the independent ethics committee of the Affiliated Hospital of Qingdao University (Qingdao, China). All subjects signed a written informed consent form, and all experiments were performed following study protocol.

### Clinical Sample Procurement

NP tissue samples were obtained from the Department of Spinal Surgery in the Affiliated Hospital of Qingdao University. NP samples were obtained from patients with disc herniation, lumbar spondylolisthesis, and lumbar spinal stenosis who were undergoing interbody fusion surgery. During the operation, we only took tissue samples from the central area of the intervertebral disc. After that, we further cut the samples to remove residual annulus fibrosus and cartilage endplate to ensure maximum purity of the nucleus pulposus. NP samples were obtained from one patient without IVDD as the normal control group and five patients with IVDD as the IVDD group. We excluded participants who had a tumor or endocrine system diseases. Based on the preoperative lumbar MRI image, Pfirrmann grades (Pfirrmann et al., 2001) were utilized to assess the degree of degeneration. One patient (Ctrl) with spinal cord injury was diagnosed with Pfirrmann I and served as the normal control (NC); three patients (NP4, NP9, and NP10) were diagnosed with Pfirrmann II/III and served as the mild IVDD (IVDD-M); and two patients (NP2, NP8) were diagnosed with Pfirrmann IV/V and served as the severe IVDD (IVDD-S).

### NP Sample Processing and Single-Cell Dissociation

The fresh NP sample at different grades was placed into the GEXSCOPE Tissue Preservation Solution (Singleron Biotechnologies) storage and transported at 2–8°C. Firstly, all samples were washed three times with Hanks Balanced Salt Solution (HBSS), cut into pieces (1–2 mm^3^), and subjected to enzymatic digestion with 2 ml GEXSCOPE Tissue Dissociation Solution (Singleron Biotechnologies) in a 15-ml centrifuge tube at 37°C constant temperature shaker for 15 min. Subsequently, cell suspension was filtered through a 40-μm sterile cell strainer (Corning). Cell suspensions were centrifuged at 500 g for 5 min at 4°C, the suspensions were discarded, and cell pellets were resuspended with 1 ml phosphate buffer saline (PBS) (HyClone). The cell suspension was incubated with 2 ml GEXSCOPE Red Blood Cell Lysis Buffer (Singleron Biotechnologies) at 25°C for 10 min. Then it was centrifuged at 500 g for 5 min, red blood cell was removed, and the suspension was resuspended with PBS. Finally, cells were counted using a TC20 automated cell counter (Singleron), and live cells were determined by trypan blue staining (Sigma). Isolated NP was directly prepared for cDNAs amplification and single-cell RNA-Seq library construction.

### Singleron MatrixTM Single-Cell RNA Sequencing

The single-cell suspension with a concentration of 1 × 10^5^ cells/ml was loaded onto the microfluidic chip. According to the manufacturer’s protocols (Singleron GEXSCOPE Single Cell RNA-seq Library Kit, Singleron Biotechnologies), the single-cell RNA-seq library was prepared, which was captured for sequencing by using an Illumina HiSeq X with 150 bp paired-end reads (Dura et al., 2019).

### Single-Cell RNA-Seq Data Processing

To quantitatively analyze the gene expression of cells, we first removed low-quality reads by fast QC, fastp, and poly-A tails, and adaptor sequences were removed by cutadapt. After quality control, raw reads were mapped to the reference genome GRCh38 (Ensembl version 92 annotation) *via* STAR (Liao et al., 2014). Gene counts and unique molecular identifier (UMI) counts were acquired by the featureCounts software. Expression matrix files for subsequent analyses were generated based on gene counts and UMI counts. Cells were filtered by gene counts between 200 and 5,000 and UMI counts below 30,000. Cells with over 30% mitochondrial content were removed. We used functions from Seurat v2.3 for dimension-reduction and clustering (Butler et al., 2018). All gene expression was normalized and scaled using NormalizeData and ScaleData.

### Dimension-Reduction and Clustering

We used principle component analysis (PCA) to analyze the top 2,000 variance genes, which were selected by FindVariableFeautres (Shaath et al., 2020). Cells were separated into three clusters by FindClusters using the top 20 principle components and resolution parameter at 1.0. For subclustering of chondrocyte types, we set the resolution at 1.2. The t-SNE algorithm was applied to visualize cells in a two-dimensional space. According to selected PC dimensions with the Seurat R package8, we performed the t-SNE analysis.

### Differentially Expressed Gene Analysis

Differentially expressed genes (DEGs) in each cell cluster of NP were selected by Seurat FindMarkers based on Wilcox likelihood-ratio test with default parameters. Genes expressed in more than 10% of the cells in a cluster and with an average log (Fold Change) of greater than 0.25 were selected as DEGs.

### Marker Gene Analysis and Identification of Cell Types

According to Seurat’s FindAllMarkers function, we determined the marker genes of each cell group relative to other cell clusters in NP by “Wilcox” (Wilcoxon rank sum test). The cell-type identity of each cluster was manually annotated with the expression of canonical markers combined with knowledge from literature. Heatmaps/dot/violin plots displaying the expression of markers used to identify each cell type were generated by Seurat DoHeatmap/DotPlot/Vlnplot.

### Enrichment and Cell Interaction Analysis

The “clusterProfiler” R package version was used to perform enrichment analysis, including Gene Ontology (GO) and Kyoto encyclopedia of genes and genomes (KEGG) analysis (Yu et al., 2012). Pathways with p_adj value < 0.05 were considered as significantly enriched. Cell–cell interaction (CCI) analyses were predicted based on known ligand–receptor pairs by Cellphone DB (Efremova et al., 2020). Based on the average log gene expression distribution for all genes across each cell type, individual ligand or receptor expression was thresholded by a cutoff. Predicted interaction pairs with *p*-value < 0.05 and of average log expression >0.1 were considered as significant and visualized by the circlize (0.4.10) R package.

### Gene Set Variation Analysis

All canonical pathways in the website of the molecular signature database (MSigDB, version 6.2) were provided by the GSEABase package (version 1.44.0). Next, we applied the gene set variation analysis (GSVA) method with default settings to assign pathway activity estimates for individual cells, as implemented in the GSVA package (version 1.30.0). To quantify the differences in pathway activity between multiple clusters, we used a generalized linear model to contrast the enrichment scores for each cell.

### Transcription Factor Analysis

Human transcription factors (TFs) were obtained from AnimalTFDB3.0 for the downstream analysis. Enrichment of the predicted targets was assessed by comparing gene expression in each cluster between different groups, and only significant TFs were selected as candidate TFs. The TF network was constructed by the SCENIC R (Aibar et al., 2017) toolkit using the scRNA expression matrix. The GENIE3 package predicted a regulatory network based on the co-expression of regulators and targets. RcisTarget and AUCell were employed to trim modules for targets and evaluate the activity of the regulatory network on all the cells, respectively.

### Monocle Analysis

The Monocle package (version 2.99.0) was used to plot trajectories to illustrate the behavioral similarity and transitions (Qiu et al., 2017). We used an expression matrix derived from Seurat to build a CellDataSet for Monocle pipeline, and partition the cells into supergroups after dimensionality reduction. A simple PPT method was applied in organizing supergroups into a tree-like trajectory. A plot cell trajectory module was used to plot the trajectory and color the cells by subcluster type.

### Statistical Analysis

Statistical analyses were performed using R packages. ANOVA or Student’s t-test with Student–Newman–Keuls *post hoc* test was performed to determine the statistical significance between differences, and *p*-value < 0.05 was considered as statistically significant. Statistical calculations were performed by SPSS 21.0. Statistical analyses were performed in GraphPad Prism (version 8.3).

## Result

### Cellular Constitution of Human NP in IVDD

**FIGURE 1:**
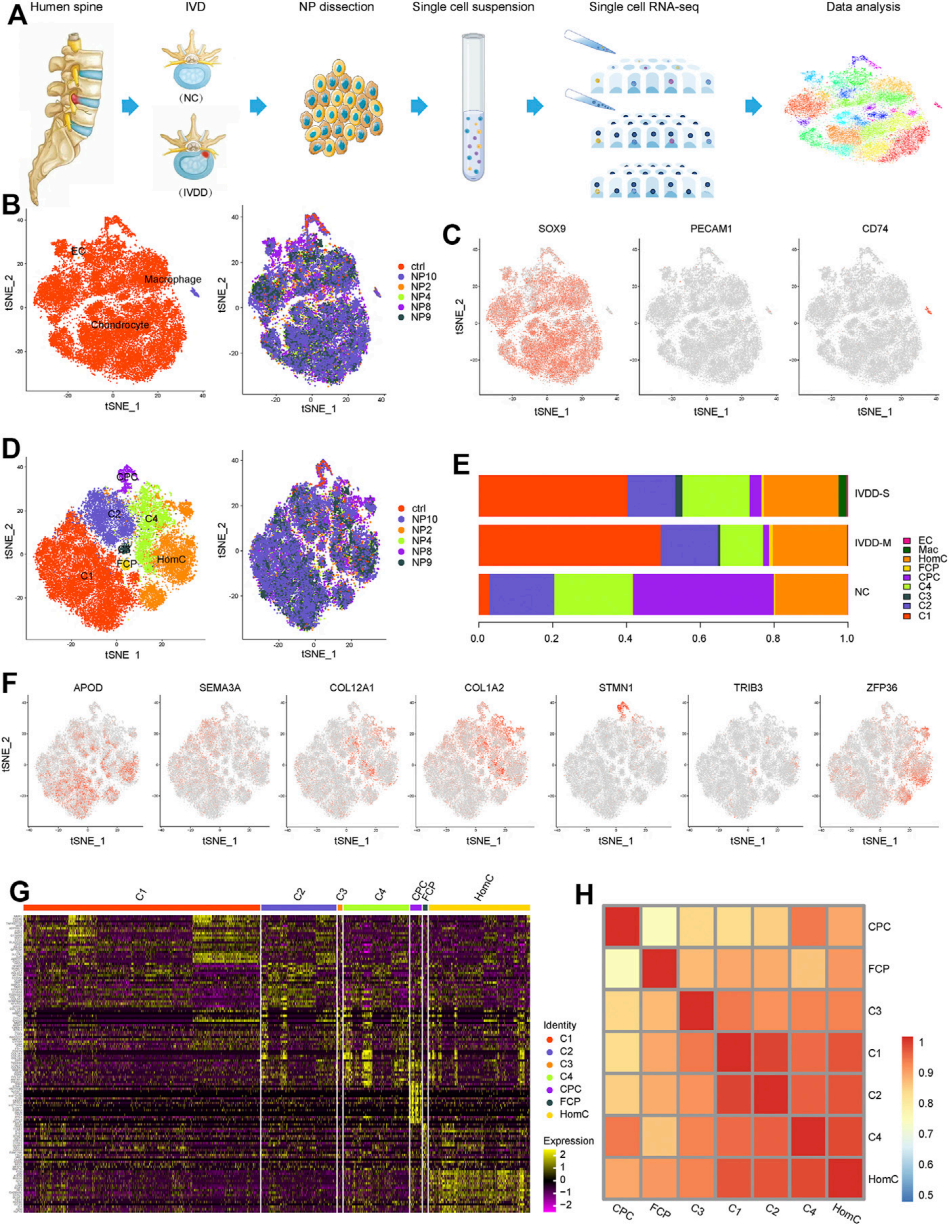
Single-cell atlas of healthy and degenerative NP samples. **(A)** Schematic diagram of sample source and experimental workflow. **(B)** t-SNE of the 30,300 cells profiled three main cell types (left) and six samples (right) distribution. **(C)** t-SNE plots showing the expression of marker genes for chondrocyte, macrophage, and EC defined above each panel. **(D)** t-SNE of the chondrocytes profiled seven subclusters (left) and six samples (right) distribution. **(E)** Bar plots represented the proportion of the distinct cell clusters in NP from NC, IVDD-M, and IVDD-S. **(F)** t-SNE plots showing the expression of marker genes for seven chondrocyte subclusters defined above each panel. **(G)** Heatmap of top 20 DEGs for seven chondrocyte subclusters; each column represents a cell cluster, and each row represents a DEG for a cluster. **(H)** Heatmap showing pairwise Pearson correlations in each chondrocyte subcluster. NP, nucleus pulposus; t-SNE, t-distributed stochastic neighbor embedding; EC, endothelial cells; CPC, cartilage progenitor cells; FCP, fibrochondrocyte progenitors; HomC, homeostatic chondrocytes; DEGs, differentially expressed genes.

To investigate cellular diversity, we used scRNA-seq analysis on six NP samples from five patients with various degrees of IVDD (IVDD-M: NP4, NP9, and NP10 and IVDD-S: NP2 and NP8) and one spinal cord injury patient without IVDD (NC) (Figure 1A). Clinical information was collected from the patient records as shown in Supplementary Table S1. A total of 30,300 cells were collected after data pre-processing and quality assessment. Each cell had an average of 6,089 UMIs and 1,671 genes. Unsupervised cell clustering revealed three major groups in parallel based on lineage-specific marker gene expression: chondrocytes (particularly expressing *SOX9*, *ACAN*, and *COMP*), endothelial cells (EC, specifically expressing *PECAM1*, *CD34*, and *PLVAP*), and macrophages (specifically expressing *CD74*, *TYROBP*, and *LAPTM*) (Figures 1B,C and Supplementary Figure S1A and Supplementary Table S2). Chondrocytes, which make up the majority of the cells in all samples, are further split into three empirically defined populations: cartilage progenitor cells (CPCs), fibrochondrocyte progenitors (FCPs), and homeostatic chondrocytes (HomCs), as well as four new populations known as C1–4 (Figures 1D,F). Figure 1F and Supplementary Figure S1B show each chondrocyte population based on cell lineage-specific marker gene expression. The top 20 differentially expressed genes (DEGs) are shown in Figure 1G and Supplementary Table S3.

Next, we compared the distribution of subclusters in each group (Figure 1E and Supplementary Table S4) and discovered that chondrocytes were the majority in the samples, whereas EC and macrophage were only slightly present in IVDD-M and IVDD-S, which is consistent with the phenomenon of inflammatory response and vascular growth in IVDD (Kwon et al., 2017; Nakazawa et al., 2018). Interestingly, we discovered that three chondrocyte subclusters are linked to the IVDD process. C1 was seldom in the NC group, whereas it was the most common chondrocyte subtype in the degenerative samples. However, as IVDD progressed, the proportion of CPCs fell dramatically. Furthermore, C3 was only found in degenerative samples, and the proportion was greater in the IVDD-S group than in the IVDD-M group.

### Characterization of Chondrocyte Subtypes

**FIGURE 2:**
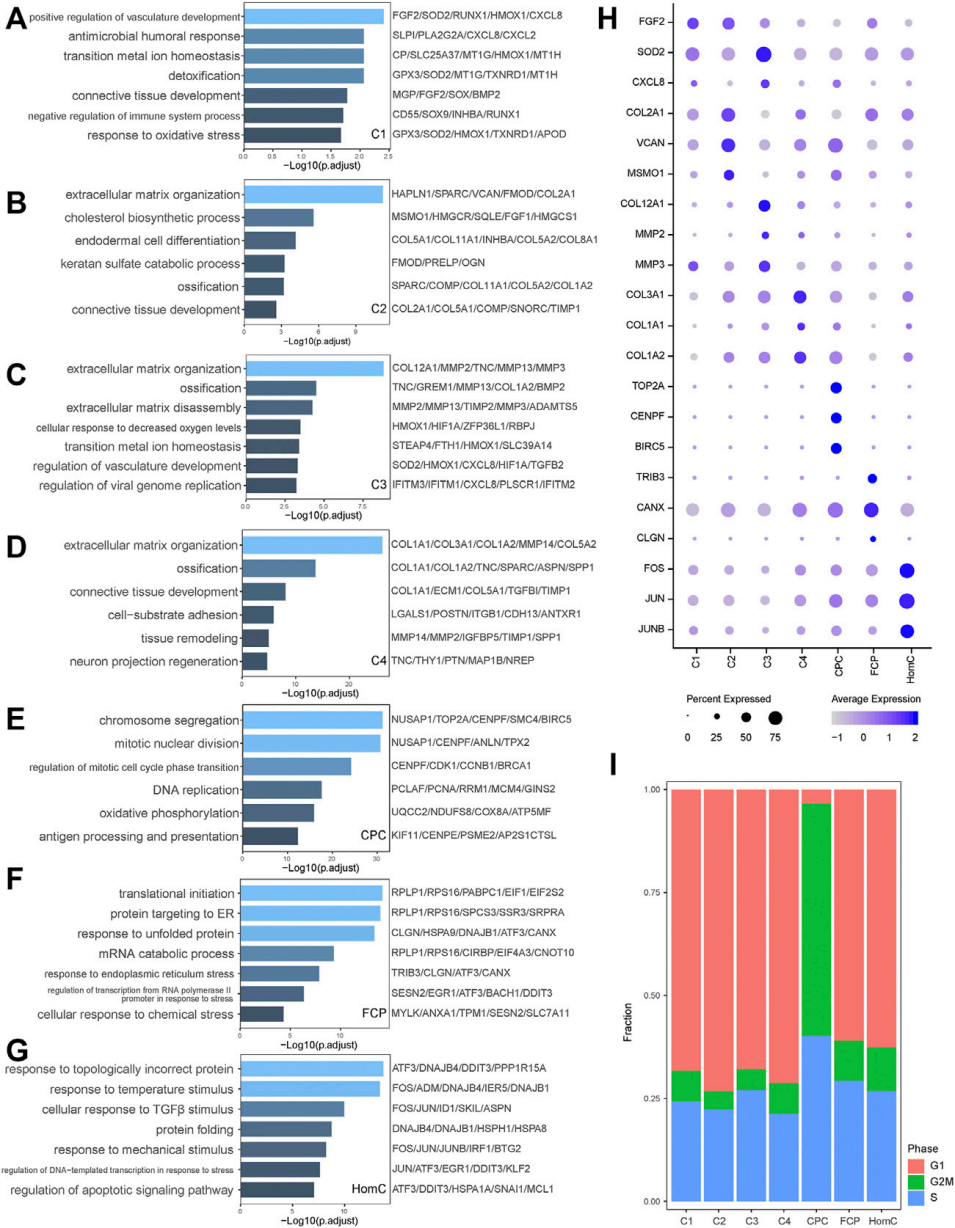
Characterization of biological functions of chondrocyte subclusters. **(A–G)** Bar plots showed the upregulated pathways and representative genes corresponding to seven chondrocyte subclusters of C1, C2, C3, C4, CPC, FCP, and HomC, respectively, by GO analysis. **(H)** Dot plot showing the expression levels of selected representative genes among seven chondrocyte subclusters. Dot size indicates the percentage of cells expressing the particular genes, and the spectrum of color represents the mean average expression of the genes. **(I)** The proportion of cells annotated as the different cell cycle phases in seven chondrocytes subclusters. CPC, cartilage progenitor cells; FCP, fibrochondrocyte progenitors; HomC, homeostatic chondrocytes.

Chondrocytes secrete ECM such as collagen and proteoglycan, which are required for the NP’s structure and function. We performed Gene Ontology (GO) analysis with DEGs to objectively comprehend the heterogeneity and biological function of chondrocyte subgroups (Figures 2A–H). Specifically, we discovered genes like *FGF2*, *HMOX1*, *CXCL8*, and *CXCL2* promote C1’s multifunctionality in the BP of positive regulation of vasculature development, antimicrobial humoral response, and transition metal ion homeostasis (Simons 2004; Semple et al., 2010; Loboda et al., 2016) (Figure 2A). Extracellular matrix organization and ossification were considerably enhanced in C2, C3, and C4, while the three chondrocytes expressed a distinct array of ECM proteins and MMPs, such as C2 expressing *COL2A1* and *VCAN*, C3 expressing *COL12A1* and *MMP2*, and C4 expressing *COL3A1* and *COL1A1*. Furthermore, the GO analysis revealed that C2 is related to the cholesterol biosynthetic process, C3 is involved in extracellular matrix disassembly, and C4 is more prone to tissue remodeling (Figures 2B–D). In summary, C1 and C3 were classified as inflammatory response subsets. C2 has a high expression of collagen type II and proteoglycan to keep the ECM’s regular structure. C4 has a phenotype similar to fibroblasts and may contribute to ECM remodeling (Reeves et al., 2014).

We also discovered that CPC, FCP, and HomC display different functions in IVDD. CPC, as NP-derived progenitor/stem cells, specifically expressed *TOP2A*, *CENPF*, and *BIRC5*, all of which are associated with chromosome segregation and mitotic nuclear division, which is consistent with our GO analysis (Bellayr et al., 2016; Kanfer and Kornmann 2016; Wheatley and Altieri 2019; Nielsen et al., 2020) (Figure 2E). Annotation of the cell cycle revealed that the fraction of CPC in the G2/M phase was substantially higher than that of other subpopulations, demonstrating its active proliferative ability (Figure 2I). FCP-related genes *TRIB3* and *CANX* have been linked to protein folding quality control and ER stress (Mondal et al., 2016; Forrester et al., 2019) (Figure 2F). HomC has a significant capacity to respond to stress, such as response to topologically incorrect protein, response to temperature stimulus, and cellular response to TGFβ stimulus. *FOS/JUN*, as transcription factors (TFs), were crucial in regulating the HomC response to stress (Wagner 2001) (Figure 2G). Above all, these seven chondrocytes perform a variety of tasks while retaining a high degree of connection (Figure 1H).

### Transcriptional Regulation in Chondrocyte Subpopulations

**FIGURE 3:**
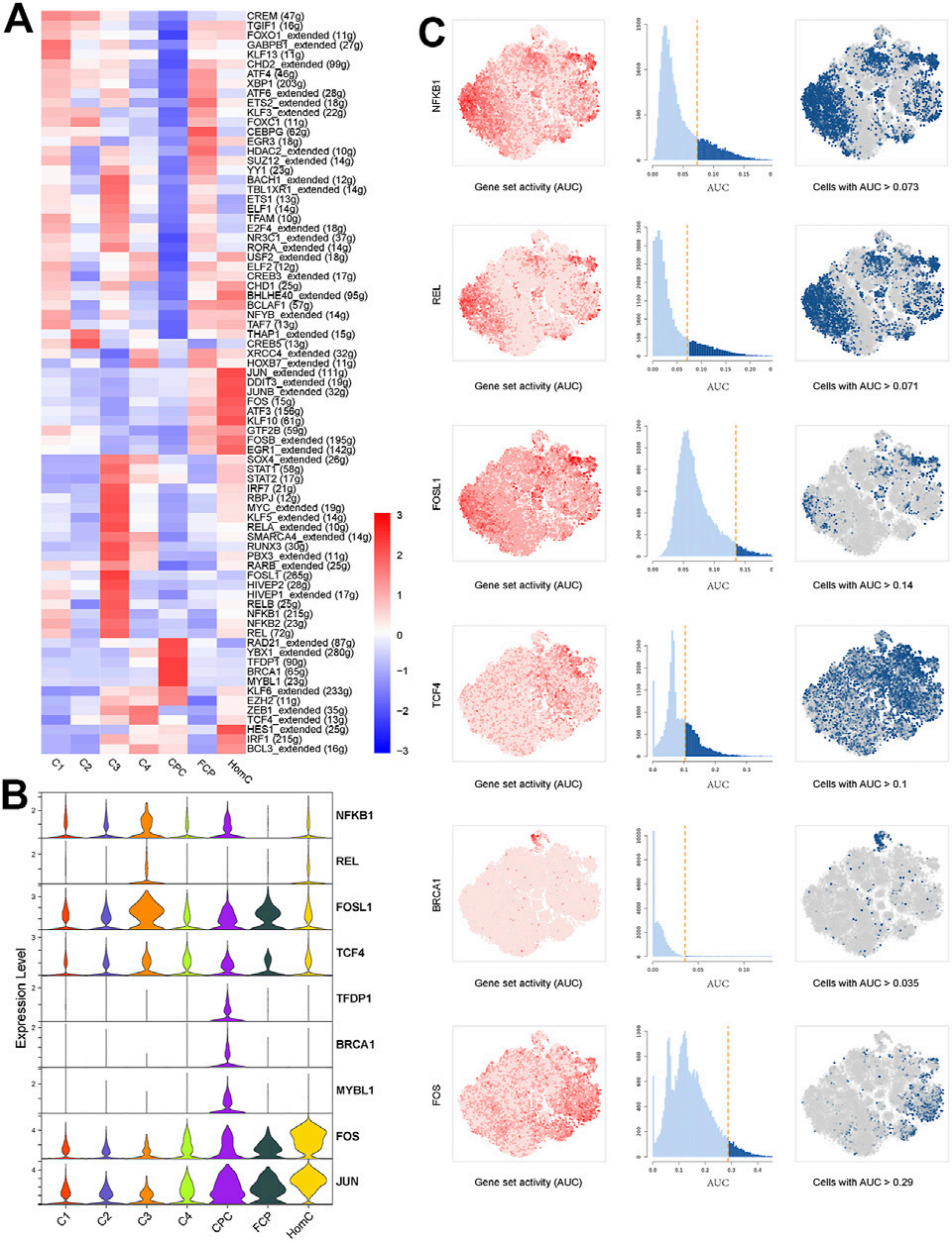
Transcriptional regulation of seven chondrocyte subclusters. **(A)** Heatmap indicating the expression regulation by TFs analyzed with SCENIC in seven chondrocyte subclusters. Numbers between brackets indicate the (extended) regulons for respective TFs. **(B)** Violin plots showing the expression levels of representative candidate TFs across the seven chondrocyte subclusters. **(C)** SCENIC analysis predicts selected TFs as central hubs governing the different chondrocyte subclusters. TF regulon activities were quantified using AUCell. TFs, transcription factors; SCENIC, single-cell regulatory network inference and clustering; CPC, cartilage progenitor cells; FCP, fibrochondrocyte progenitors; HomC, homeostatic chondrocytes.

The single-cell regulatory network inference and clustering (SCENIC) approach was performed to investigate the TFs that may govern various chondrocyte phenotypes. This analysis revealed that several TF networks were enriched in distinct subclusters (Figure 3A). In C1 and C3, the NF-kappaB family (*NFKB1*, *NFKB2*, and *REL*) shows enhanced regulatory activity, which is consistent with the inflammatory response subgroups, but *FOSL1* and its target genes, including *MMP2*, *MMP3*, and *MMP13*, are substantially upregulated in C3 (Chen et al., 2020). *TCF4* regulon activity is greater in C4, which may contribute to C4’s fibrous characteristics (Mathew et al., 2011). *TFDP1*, *BRCA1*, and *MYBL1* are CPC-specific regulons that play important roles in the cell cycle and DNA damage repair control (Qiao et al., 2007; Wu et al., 2010; Xie et al., 2020). Coincidentally, *TFDP1* controls *BRCA1* transcriptional activity in chondrocytes (Pellicelli et al., 2016). Furthermore, SCENIC results revealed that the regulatory activities of *JUN* and *FOS* regulons in HomC were highly elevated, which is consistent with GO analysis (Figures 3B,C and Supplementary Figure S1C). Thus, we investigated the potential upstream regulatory TFs, which will aid in our understanding of the heterogeneity of NPCs and their involvement in IVDD.

### The Trajectory of Chondrocytes in NP

**FIGURE 4:**
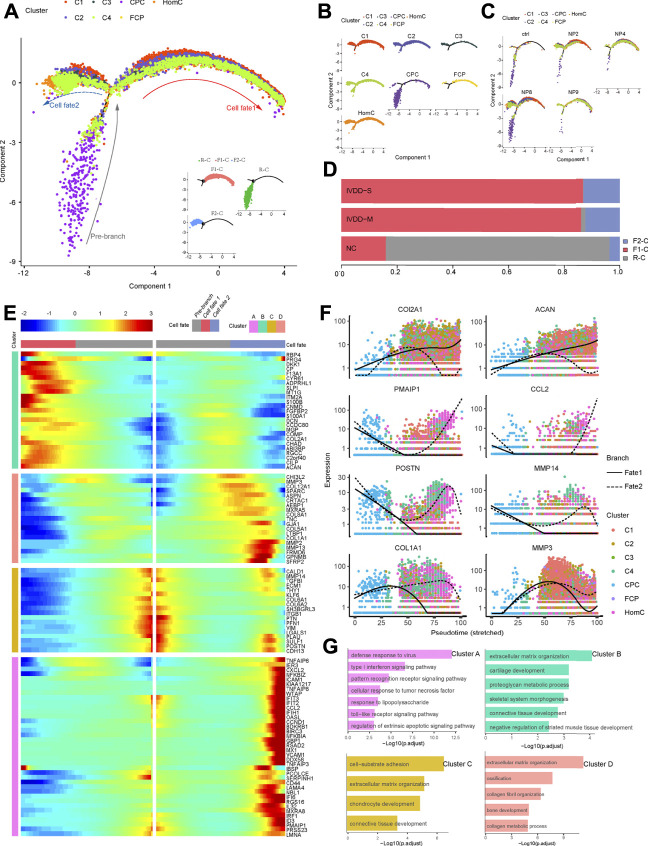
Pseudotime trajectory analysis depicted chondrocyte fate differentiation in IVDD. **(A)** Monocle two trajectory plot contains three branches and one connecting point showing dynamics of chondrocyte subclusters (except NP10). **(B,C)** Projection of individual cell types and samples onto the trajectory of **(A)**. **(D)** Bar plots represented the proportion of different cell states corresponded to three branches from NC, IVDD-M, and IVDD-S. **(E)** Heatmap showing the standardized kinetic curves of the genes in trajectory from root to fate1 or fate2. **(F)** Pseudotime kinetics of specific representative genes from the root of the trajectory to fate1 (solid line) and fate2 (dashed line). **(G)** Bar plots showing the top annotated GO terms in four genes sets, which are hierarchically clustered from **(E)**. CPC, cartilage progenitor cells; FCP, fibrochondrocyte progenitors; HomC, homeostatic chondrocytes.

Changes in chondrocyte expression patterns are closely linked to the development of IVDD (Li et al., 2019; Tsingas et al., 2020). To study the conversion of chondrocytes in NP during IVDD development, we performed a trajectory analysis of various chondrocyte subtypes by Monocle two method in all samples (Supplementary Figure S2A). However, we discovered that NP10 is not suitable for the current analysis programs because its pseudotime trajectory did not fit well enough with other samples from the IVDD-M group, which could be due to the presence of degenerative spondylolisthesis and the “intervertebral disc vacuum phenomenon” at the sample segment in this patient (Supplementary Figure S2B). Then, except for NP10, we ran a trajectory analysis on chondrocytes and created a pseudotime trajectory axis with two terminals corresponding to two distinct cell fates (Figures 4A–C). The cells in the root of the trajectory were labeled R-C, the cells in fate1 were labeled F1-C, and the cells in fate2 were labeled F2-C (Figure 4A). In the pseudotime trajectory, we compared the dispersion patterns of several chondrocyte subtypes. CPC is mostly found near the root. C1 mostly follows two fates, C3 and FCP primarily occupy the ends of the two fates, and C2, C4, and HomC are scattered along the trajectory (Figure 4C). Figure 4D revealed that the proportion of R-C progressively dropped, while the proportion of F1-C and F2-C steadily rose as the disease progressed. In general, our result indicates that the distribution of chondrocyte subsets in the chondrocyte fate differentiation curve was consistent with the IVDD pathological process.

Next, using the results of the trajectory analysis, we deconstructed sample gene transformation patterns to investigate the precise impact of the alterations in cell fate (Figure 4E). These genes were divided into four distinct clusters. Cluster “A” enriched with genes (*PMAIP1*, *CCL2*, *CXCL2*, and *ICAM1*) linked to defense response to virus, type I interferon signaling pathway, and cellular response to TNF was dramatically elevated in the transition to fate2. Meanwhile, clusters C and D genes enriched in the ECM structure are also expressed in the direction of fate2, including *MMP2*, *MMP3*, *POSTN*, and *COL1A1*. Cluster “B” genes (*COL2A1* and *ACAN*) highly expressed in normal ECM were dramatically enhanced in the transition to fate1 (Figures 4E–G).

Finally, trajectory analysis revealed that during IVDD, NPC differentiated into two cell fates (R-C to F1-C and R-C to F2-C). The process of chronic inflammation and changes in ECM has a high correlation with the two cell fates, implying that studying the differentiation direction of NPCs, particularly the mechanism network of fate2, may provide an effective strategy for the treatment of IVDD.

## The Different Cell Fate Determination of NPCs Reflects a Landscape of IVDD Development

**FIGURE 5:**
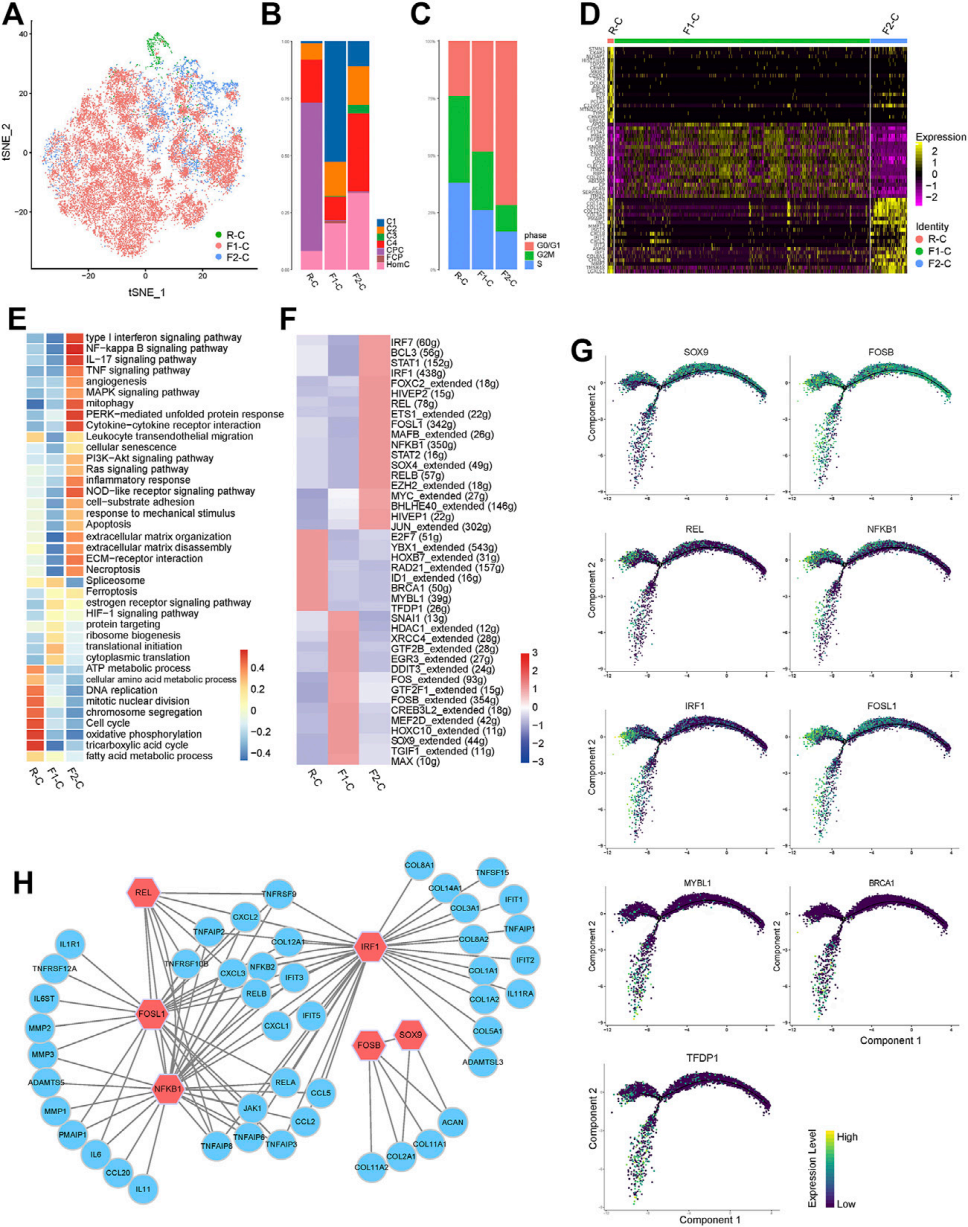
Transcriptional regulation related to chondrocyte differentiation fate. **(A)** t-SNE of the chondrocytes profiled three cell states (R-C, F1-C, and F2-C). **(B)** Bar plots represented the proportion of seven chondrocyte subclusters in R-C, F1-C, and F2-C. **(C)** Bar plots represented the proportion of cells annotated as the different cell cycle phases in R-C, F1-C, and F2-C. **(D)** Heatmap showing top 20 DEGs for R-C, F1-C, and F2-C; each column represents a cell state, and each row represents a DEG for a state. **(E)** The heatmap of GSVA of the GO and KEGG gene sets related to IVDD among the three cell states. **(F)** Heatmap indicating the expression regulation by TFs analyzed with SCENIC in three cell states. Numbers between brackets indicate the (extended) regulons for respective TFs. **(G)** The expression levels of the selected TFs on pseudotime trajectories. **(H)** Regulatory networks consisting of selected TFs and their target genes drive cell fate differentiation. A node represents a gene and edges represent the interactions. Red nodes are hub TFs. CPC, cartilage progenitor cells; FCP, fibrochondrocyte progenitors; HomC, homeostatic chondrocytes; DEGs, differentially expressed genes; TFs, transcription factors; SCENIC, single-cell regulatory network inference and clustering.

To deeply insight into the mechanism behind IVDD, we utilized GSVA to characterize R-C, F1-C, and F2-C functions, and SCENIC to discover candidate TFs. Figures 5A–D and Supplementary Table S5 depict more precise information about cell distribution, cell cycle annotation, and DEGs from chondrocyte cells in three stages.

R-C is primarily concentrated in the cell cycle and oxidative phosphorylation (Figure 5E). However, when the cells are differentiated into the two cell fates, the expression of TFs (*TFDP1*, *BRCA1*, and *MYBL1*) is reduced (Figures 5F,G). F1-C cells are primarily enriched in protein targeting, translational initiation, and ribosome biogenesis (Figure 5E), indicating that these cells are primarily employed for protein synthesis, processing, and transport. Notably, the ECM produced at this stage was mostly comprised of collagen type II and proteoglycan (coded by *COL2A1* and *ACAN*), with minimal expression of *MMPs* and *ADAMTs* (Figure 5D, Supplementary Table S5). We also discovered that *FOSB* and *SOX9*, the particular transcription factors predicted by SCENIC to control *COL2A1* and *ACAN*, were significantly expressed in this condition (Figures 5F–H). F2-C genes were enriched in an ECM organization and ECM disassembly, such as *COL1A1*, *COL1A2*, *COL3A1*, and *MMP13*. Meanwhile, F2-C highly expressed a wide range of inflammatory chemokines, including *CXCL8* and *CXCL2*. Furthermore, F2-C overexpressed several interferon-induced genes, including *IFIT1*, *IFIT2*, and *IFIT3*. Meanwhile, *JAK1* and *STAT1*, which are upstream regulatory components of interferon-induced genes, were also considerably increased (Zhou et al., 2013) (Figures 5D,E and Supplementary Table S5). The interferon signaling pathway is widely recognized to have antiproliferation and pro-apoptotic effects, which may be one of the reasons for the reduction in the number of NPCs in IVDD. We discovered that *NFKB1*, *FOSL1*, *IRF1*, and *REL* have strong regulatory activity and can control each other at this stage using SCENIC, and interestingly, the predicted target genes of these TFs are the same genes that are highly expressed in F2-C. Furthermore, TNF, NF-κB, MAPK, IL17, and type I interferon signaling pathways enriched in F2-C can cause the upregulation or activation of these TFs (Beg and Baldwin 1994; Wang et al., 2017; Bonelli et al., 2019) (Figures 5E–H). Taken together, these data indicated TFs and signal pathways that govern the fate of various cells in IVDD, contributing to a better understanding of the disease mechanism.

### Constructing a Cell Fate-Based Cell Communication Network for IVDD

**FIGURE 6:**
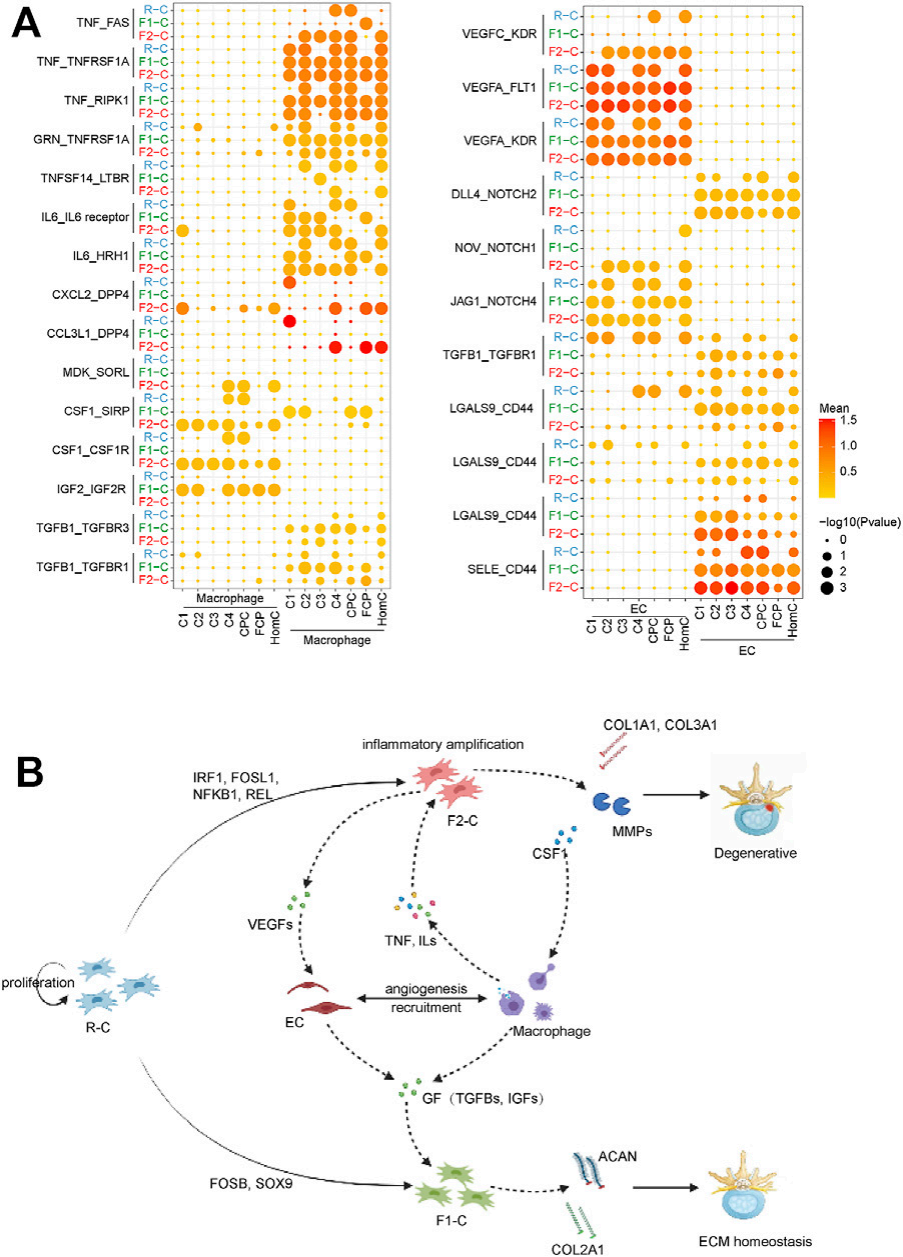
Network of regulatory mechanisms for IVDD. **(A)** Dot plots depicting ligand–receptor pairs between chondrocytes and macrophage (left) and EC (right). Dot size indicates the percentage of cells expressing the particular genes and the spectrum of color represents the mean average expression of the genes. **(B)** Diagram of the regulatory mechanisms of IVDD found in this study. Solid arrows represent the direction of cell differentiation; dashed arrows represent cytokines and extracellular matrix secreted by cells.

The ligand–receptor pair facilitates cell contact, influences cell function and destiny, and promotes or inhibits disease development. In our study, macrophages and EC were only found in the IVDD group, and we utilized CellPhoneDB to investigate potential connections between chondrocytes and macrophages and EC at various cell fates (Figure 6A).

Macrophages secrete a large number of TNF ligand superfamily cytokines, including *TNF*, *TNFSF10*, and *TNFSF14*, whereas chondrocytes express corresponding receptors, including *LTBR*, *TNFRSF1A*, *RIPK1*, and *FAS*, indicating that the macrophage plays an apoptosis-mediated role in IVDD via the TNF signaling pathway. Surprisingly, our findings revealed that FAS-mediated programmed cell death favors F2-C (Wallach et al., 1999). Meanwhile, F1-C inhibits TNF signal-mediated apoptosis via *GRN* binding to TNF signaling receptors (Tang et al., 2011). Macrophages produce *CCL3L1* and *CXCL12*, which bind to the DPP4 receptor expressed by chondrocytes. *DPP4* is overexpressed in osteoarthritic chondrocytes to have a pro-inflammatory impact (Wang et al., 2020). F2-C, in turn, controls macrophage proliferation, migration, and differentiation via *CSF1* and *MDK* (Sato et al., 2001; Lin et al., 2019). This reveals the interplay between F2-C and macrophages in inflammatory amplification. GFs are cytokines that control cell proliferation and differentiation. Our findings suggest that F1-C interacts more with macrophages *via* GFs (*TGFB1*, *IGF2*). Recent research has demonstrated that the TGF pathway promotes *SOX9* production in chondrocytes (Lee et al., 2008), which helps to explain why *SOX9* is so abundant in F1-C (Figure 6A).

Chondrocytes express vascular endothelial growth factors (VEGFs), which include *VEGFA*, *VEGFB*, and *VEGFC*, the most potent angiogenesis agents (Dai and Rabie 2007). VEGFs, as ligands, can stimulate EC proliferation, migration, and differentiation by binding to EC-expressed *KDR* and *FLT1*. The connection between chondrocytes and EC is also mirrored in the Notch family’s control of cell destiny (including *NOTH1*, *NOTH2*, and *NOTH4*) (Ramasamy et al., 2014), and these interactions are more visible in F2-C. Our findings revealed that R-C chondrocytes influenced EC via TGF-β, whereas EC influenced F1-C chondrocytes via TGF-β. Furthermore, *SELE* and *LGALS9*-expressing EC interact with the ligand *CD44* produced by chondrocytes, which plays a crucial role in the regulation of cell contact and cell adhesion (Auguste et al., 2007; O'Brien et al., 2018) (Figure 6A).

Finally, examination of intercellular communication indicated signal crosstalk between chondrocytes, macrophages, and EC. F1-C was more active in the GF pathway, which can maintain the normal development of the chondrocytes. In contrast, in F2-C, macrophages and ECs interact with chondrocytes to promote chondrocyte apoptosis and enhance inflammatory response, which may be a key factor for the development of IVDD.

## Discussion

NPCs are a crucial functional component of the IVD, and thorough knowledge of their transcriptome profile in degeneration will aid in the development of innovative treatment methods. In this study, we found out several intriguing discoveries by doing single-cell sequencing on NPCs from different phases of IVDD.

Adults believe the healthy IVD to be an immune privilege, an avascular organ whose nutrient supply is largely dependent on fluid diffusion from the CEP (Urban et al., 2004). IVDD is an unavoidable aging process that involves an inflammatory response and subsequent ECM remodeling (Le Maitre et al., 2007; Chen et al., 2017; Tsingas et al., 2020), which is consistent with our results that macrophages and ECs were exclusively detected in the degenerative NP. The uneven distribution of macrophages and EC between IVDD-M and IVDD-S indicates that they play a role in the IVDD process.

Earlier research has demonstrated that the NP develops from the notochord during embryonic development (Lawson and Harfe 2015). As a result, we looked at the expression of the notochord cell markers *KRT8*, *KRT18*, *KRT19*, *FOXA2*, and *TBXT* in chondrocytes (Weiler et al., 2010) (Supplementary Figure S2C). Although these cells are sporadic and not clustered by the t-SNE, this result confirms earlier findings that as the NP develops, notochord lineage cells gradually vanish in the IVD, but a small number of cells continue to express notochord cell markers, at the same time indicating the dynamic evolution of the NP phenotype (Stosiek et al., 1988; Weiler et al., 2010).

In this study, we identified three empirically defined and four novel chondrocyte subsets. Our findings revealed that the quantity of C1 and C3 rose significantly in the IVDD group and that they were mostly dispersed in two branches of cell destiny in the trajectory tree. Furthermore, C1 and C3 had greater levels of chemokines (*CXCL8* and *CXCL2*) and matrix-degrading enzymes (*MMP2*, *MMP3*, and *MMP13*), all of which are thought to be important mediators of the inflammatory response. Interestingly, C1 and C3 are also abundant in transition metal ion homeostasis, with genes *CP*, *Hmox1*, and *STEAP4* playing key roles in iron and copper ion transport and redox processes. According to Jomova and Valko (2011), disturbance of transition metal ion homeostasis can cause oxidative stress and increased generation of reactive oxygen species, which can contribute to the onset of a variety of diseases, including chronic inflammation. Our previous research found that ferroptosis occurred in NPCs and accelerated IVD degeneration (Zhang et al., 2021). However, the underlying mechanism of ion-homeostasis system in IVDD needs to be investigated further. In summary, the functional study of seven chondrocyte subgroups offered more thorough knowledge of the maintenance and control of NP homeostasis by elucidating NP cell heterogeneity.

Significant research has provided insight into the ability of mesenchymal stem cells to heal damaged intervertebral discs (Richardson et al., 2016). Despite the existence of progenitor cells developing into osteogenic, adipogenic, or chondrogenic lineages in degenerative adult IVD discovered in a previous work by Risbud et al. (2007), the monitoring of the resident progenitor cells in the NP has remained a mystery until now. Recently, a collection of single-cell sequencing data on articular cartilage was used to identify the existence of CPC (Ji et al., 2019). Based on our findings, CPC was plentiful in normal IVD and steadily reduced with degeneration, and has a high proliferative capacity and greater energy consumption. Above all, the findings of this study are likely to pave the way for a novel strategy to treating IVDD utilizing NP-derived progenitor cells.

Through pseudotime analysis, we established the trajectory of chondrocyte development toward two cell fates. During the degeneration process, we witnessed chondrocytes transforming into two opposing cell phenotypes. SCENIC revealed the major regulatory function of *FOSB* and *SOX9* for *COL2A1* and *ACAN* from R-C to F1-C (fate1). Furthermore, CellphoneDB indicated that chondrocytes have a higher contact with macrophages and EC in this phase via TGF-β signaling pathways. Previous research has shown that *SOX9* stimulates NPC formation and maintains ECM homeostasis (Cucchiarini et al., 2007; Lefebvre and Dvir-Ginzberg 2017; Tsingas et al., 2020). Interestingly, recent studies have shown that the TGF-β signal pathway can increase the transcriptional level of *COL2A1* and *ACAN* by upregulating the expression of *SOX9* (Lee et al., 2008; Bozhokin et al., 2020), which explains why *COL2A1* and *ACAN* are abundant at this phase.

On the other hand, our findings show that chondrocyte differentiation to fate2 is the primary factor causing IVDD. Chen et al. (2020) found that *FOSL1* can regulate the expression of *MMPs* to promote the migration of decidua stromal cells. Forero et al. (2019) showed that IRF1 is activated by type I interferons and upregulates the expression of multiple inflammatory factors. Campbell et al. (2000) reported that *NFKB1* and *REL* are essential for mediating inflammatory responses in rheumatoid arthritis. Our data establish that these TFs (*NFKB1*, *IRF1*, *REL*, and *FOSL1*) increased the production of collagen, inflammatory factors, and matrix-degrading enzymes, all of which are linked with ECM remodeling in IVDD. CellphoneDB research indicated that F2-C interacts with macrophages and EC, causing inflammation to be amplified. Macrophages boost F2-C’s inflammatory response by secreting *IL6* and *TNF*, whereas F2-C’s *CSF1* stimulates macrophage proliferation, differentiation, and migration. Furthermore, *VEGFs* are abundantly expressed in F2-C, where they stimulate EC proliferation, migration, and differentiation while also playing an essential role in the development of microvessels, which can speed up macrophage recruitment (Figure 6B). Thus, our findings indicate that cells from the same subpopulation produced by single-cell clustering have distinct fates in practice. However, the precise causes causing this occurrence must be investigated and debated further.

In conclusion, our single-cell sequencing data provided a detailed inventory of the NPCs in IVDD from a single-cell perspective. Our data corroborated earlier findings and presented fresh study paths with potential therapeutic targets by identifying critical cell subclusters, signal pathways, and TFs, modeling cell–cell interactions, and, most significantly, giving insights into cell fate determination.

## Data Availability

The data presented in the study are deposited in the China National GeneBank repository, accession number CNP0002664 (https://db.cngb.org/search/project/CNP0002664/).
