# Supplementary material for: Single-Cell RNA Sequencing of the Nucleus Pulposus Reveals Chondrocyte Differentiation and Regulation in Intervertebral Disc Degeneration
Source: Front Cell Dev Biol. 2022 Feb 21;10:824771. doi: 10.3389/fcell.2022.824771 (PMC8899542; doi:10.3389/fcell.2022.824771)
Supplement: Supplementary file 5 [file DataSheet1.DOCX]

Supplementary Material

# Supplementary Figures and Tables

## Supplementary Figures

**
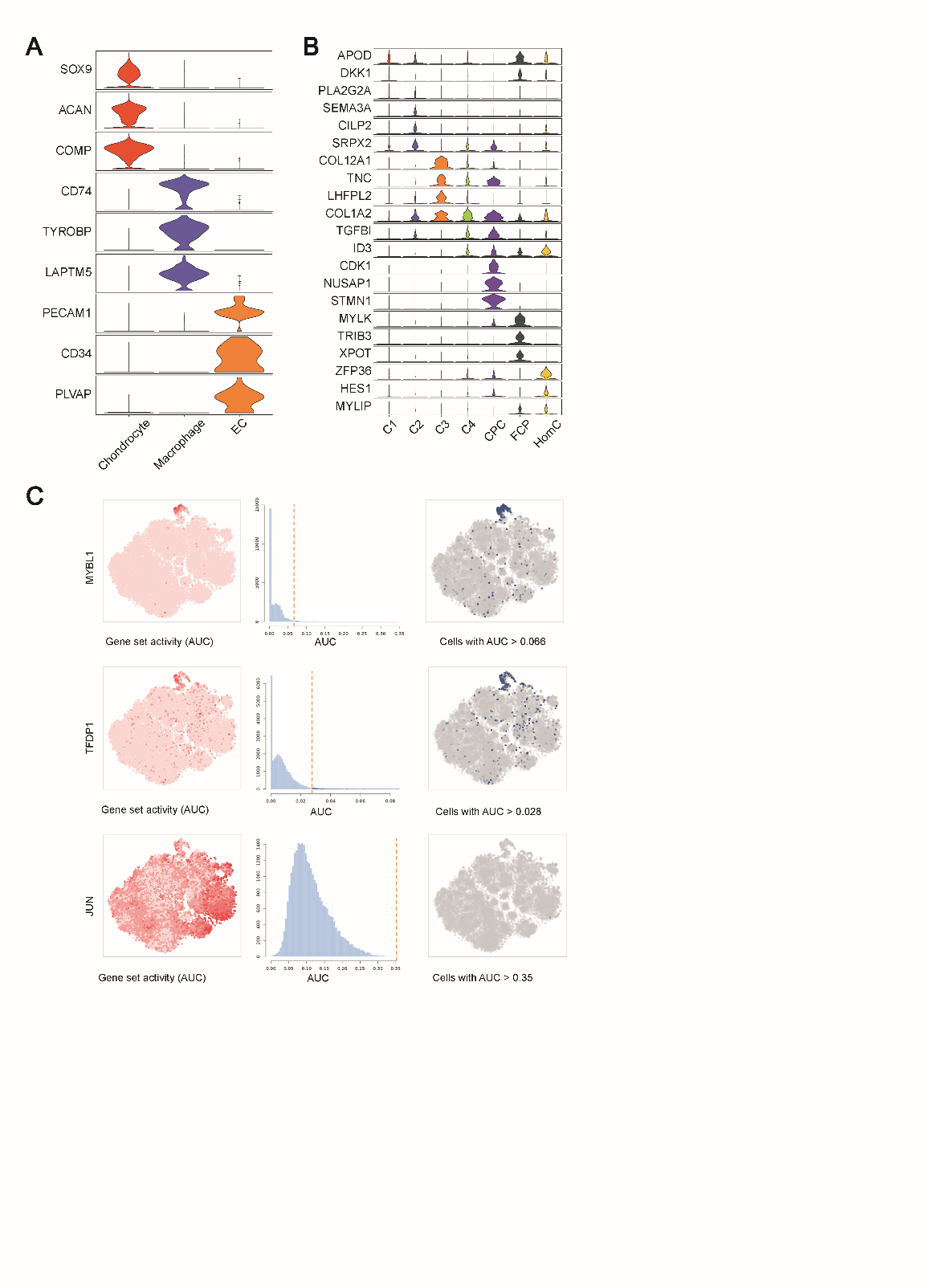
**

**Supplementary Figure 1.** Cell marker and Regulon activity. (A) Violin plots showing the expression of marker genes for chondrocyte, macrophage and EC. (B) Violin plots showing the expression of marker genes for 7 chondrocyte subclusters. (C) TF regulon activities were quantified using AUCell. CPC, cartilage progenitor cells; FCP, fibrochondrocytes progenitors; HomC, homeostatic chondrocytes; EC, endothelial cells; TF, Transcription factor.

**
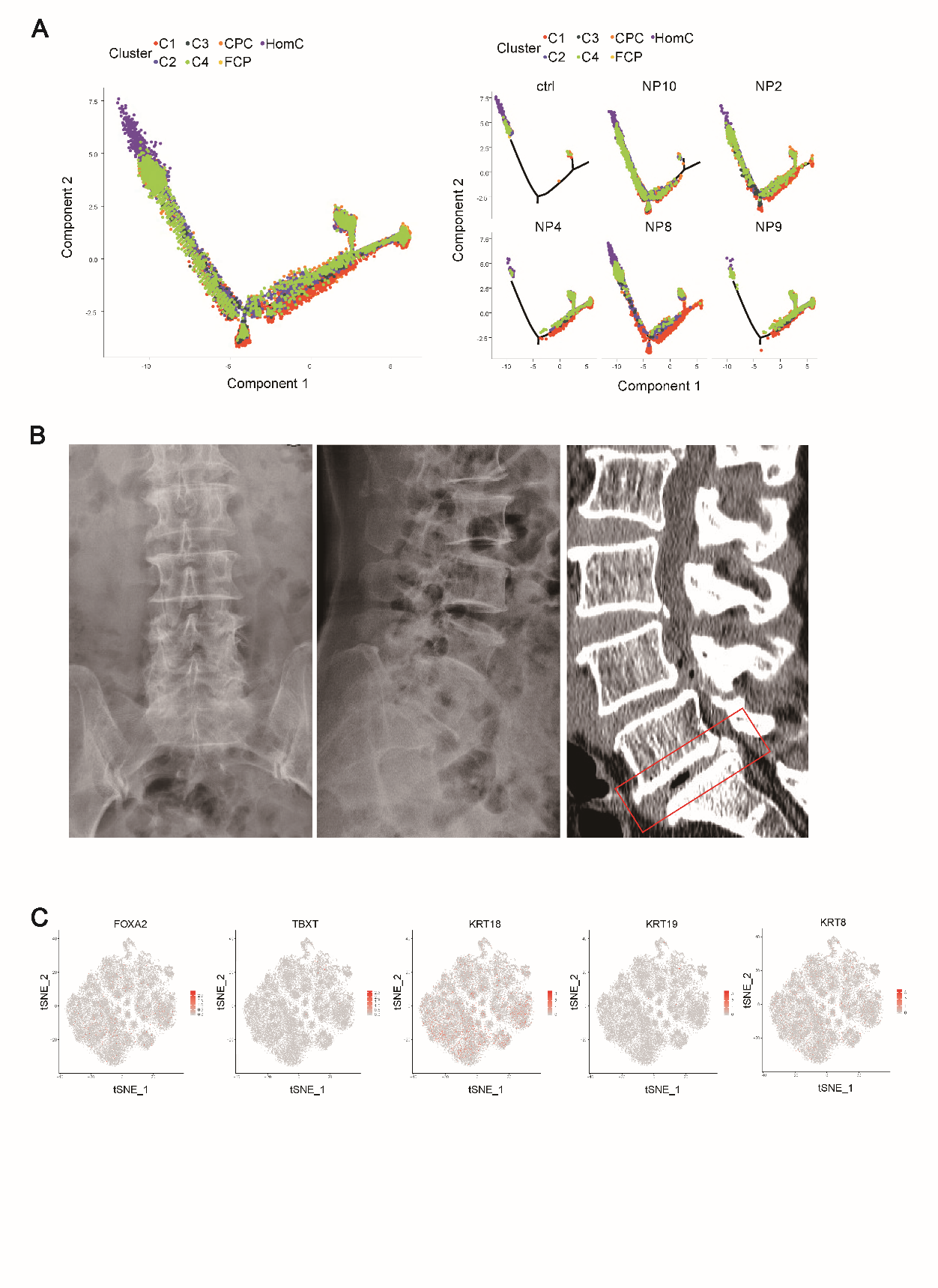
**

**Supplementary Figure 2**. Pseudotime trajectory and NP10 preoperative imaging and notochord cell marker genes. (A) Monocle 2 pseudotime trajectory showing dynamics of all chondrocyte subclusters. NP10 not fit well enough with other samples from the IVDD-M group (NP4, NP9). (B) Lumbar X-ray (left, middle) and CT (right) images showed degenerative spondylolisthesis and “intervertebral disc vacuum phenomenon” at the L5/S1 level of NP10. (C) t-SNE plots showing the expression of notochord cell marker genes in chondrocytes.

## Supplementary Tables

**Supplementary Table 1.** Detailed information of samples collected in the study.

**Supplementary Table 2.** DEGs among the 3 major clusters defined in NP.

**Supplementary Table 3.** DEGs among the 7 chondrocyte subpopulations defined in NP.

**Supplementary Table 4.** The cell number and proportion of the distinct cell clusters in NP from NC, IVDD-M and IVDD-S.

**Supplementary Table 5.** DEGs among the R-C, F1-C, and F2-C in pseudotime trajectory.
